# Supplementary material for: Exaggerated Autophagy in Stanford Type A Aortic Dissection: A Transcriptome Pilot Analysis of Human Ascending Aortic Tissues
Source: Genes (Basel). 2020 Oct 13;11(10):1187. doi: 10.3390/genes11101187 (PMC7650806; doi:10.3390/genes11101187)
Supplement: Supplementary file 1 [file genes-11-01187-s001.zip › supplementary.docx]

Figure S1


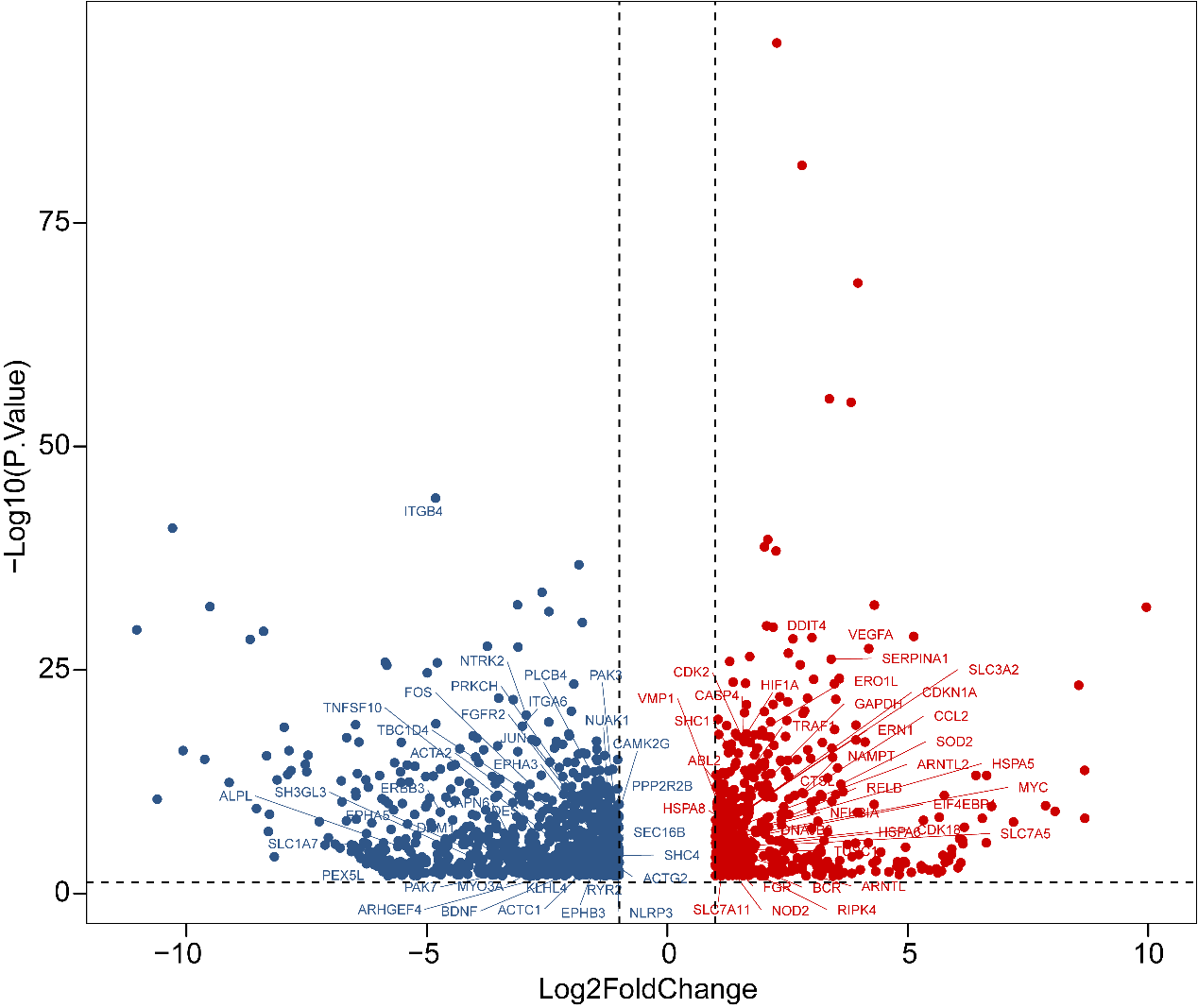


Volcano plot, all differentially expressed autophagy-related genes (DEARGs) were labeled in the plot.

Figure S2


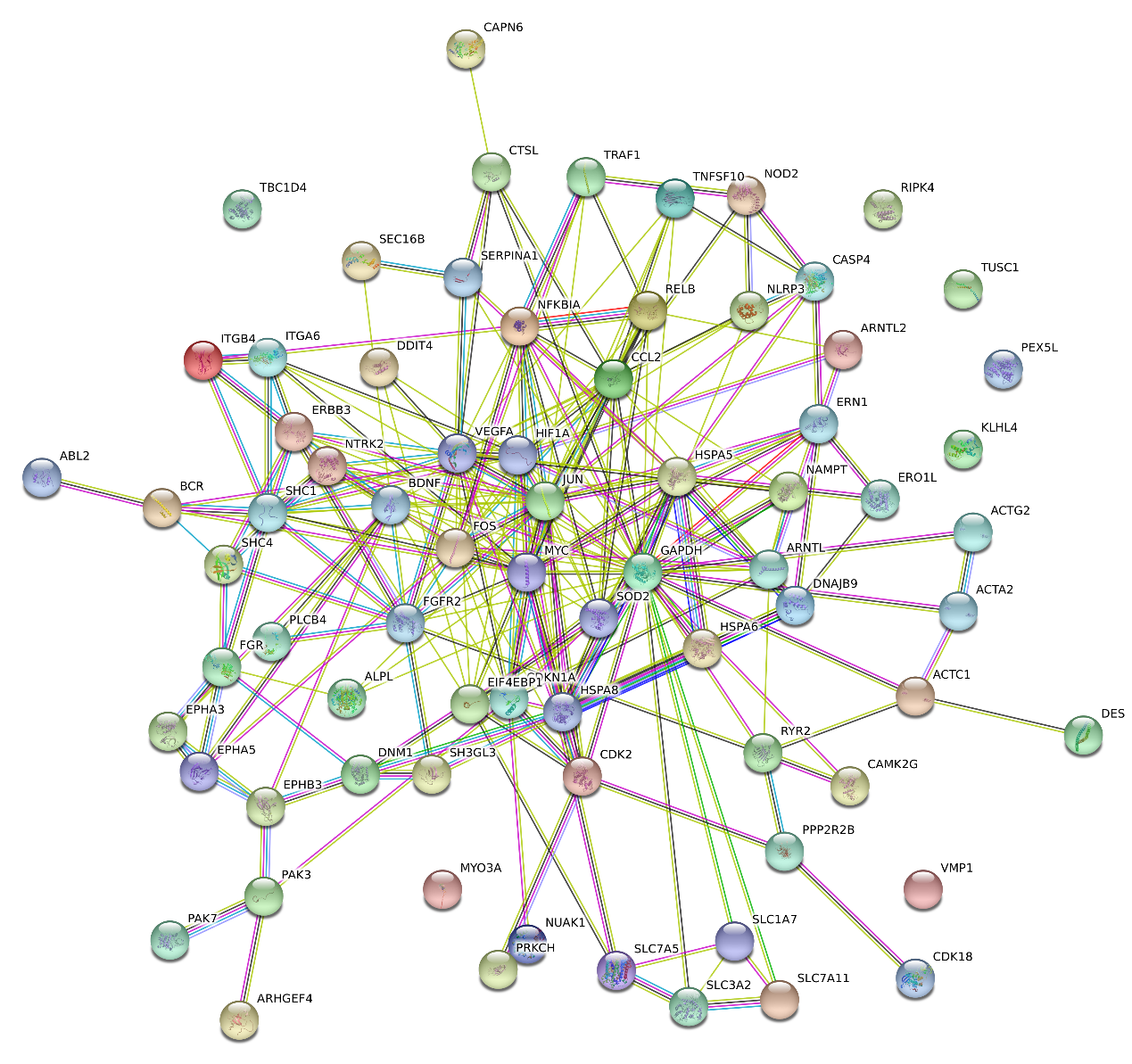


The same PPI network as Figure 5A with lager version.

Figure S3


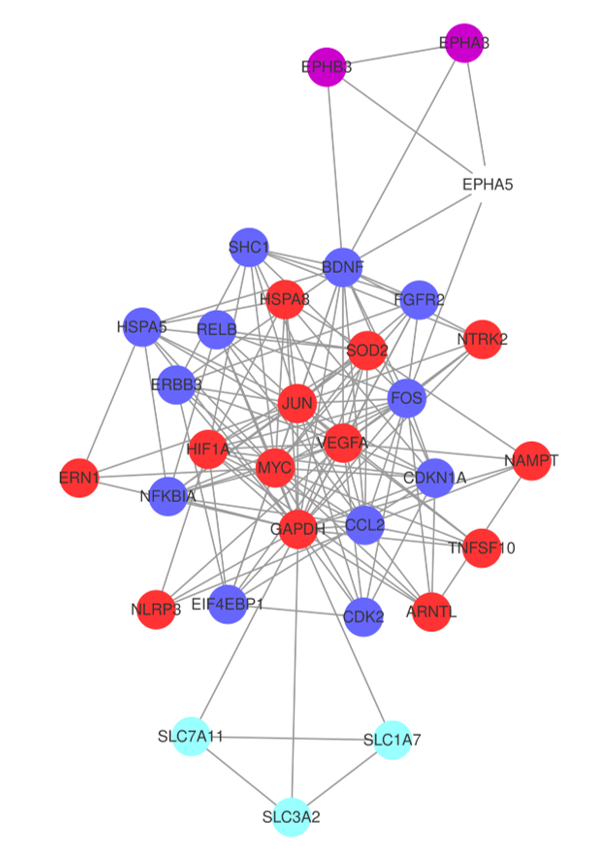


Gene modules and related genes, the same as Figure 5B with lager version. Blue: Module One; Red: Module Two; Cyan-blue: Module Three; Purple: Module Four.

Figure S4


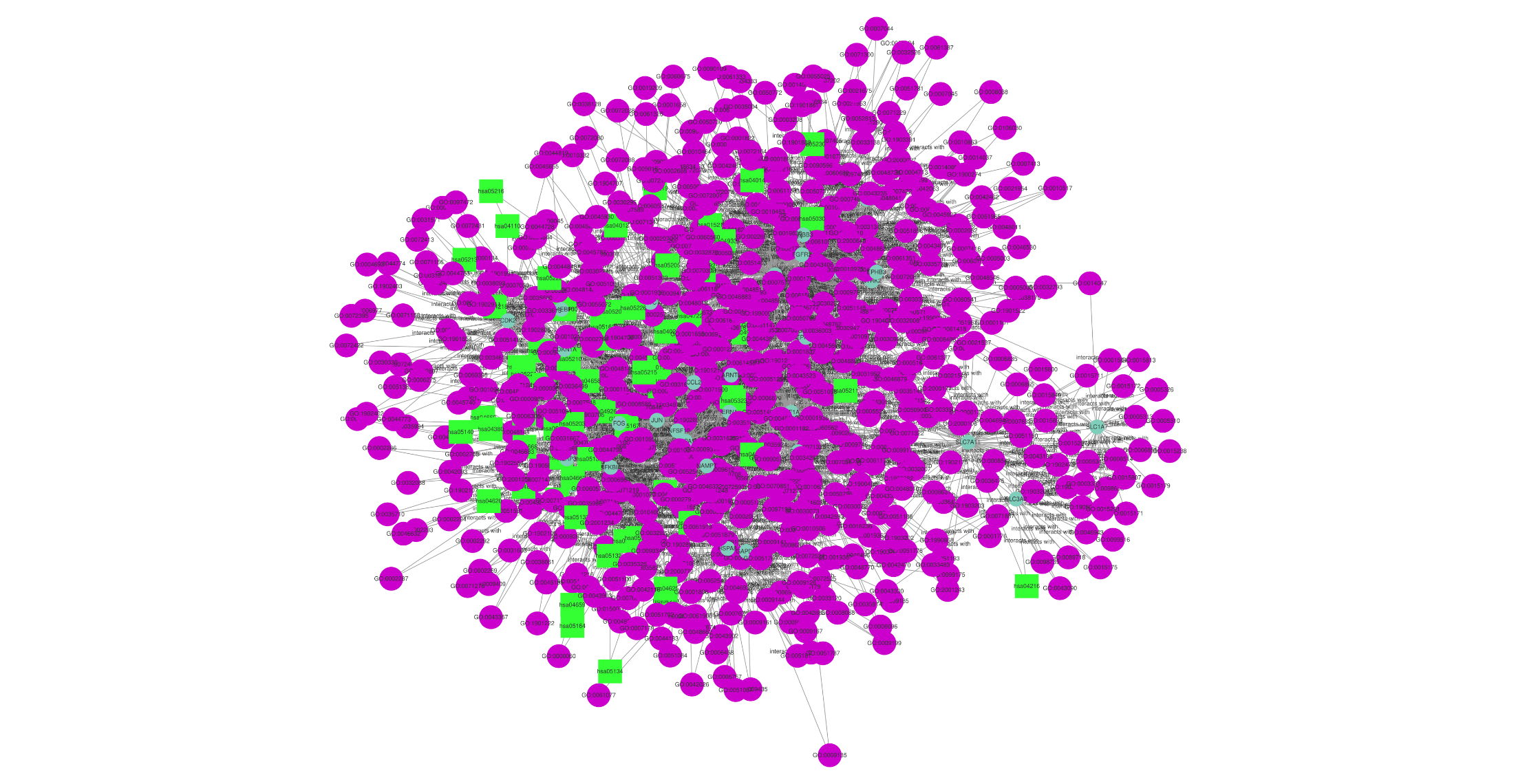


Crosstalk between gene function and signaling pathway, the same as Figure 5E with lager version. The green circles represent genes, the purple circles represent GO terms, and the green boxes represent KEGG terms.

Figure S5


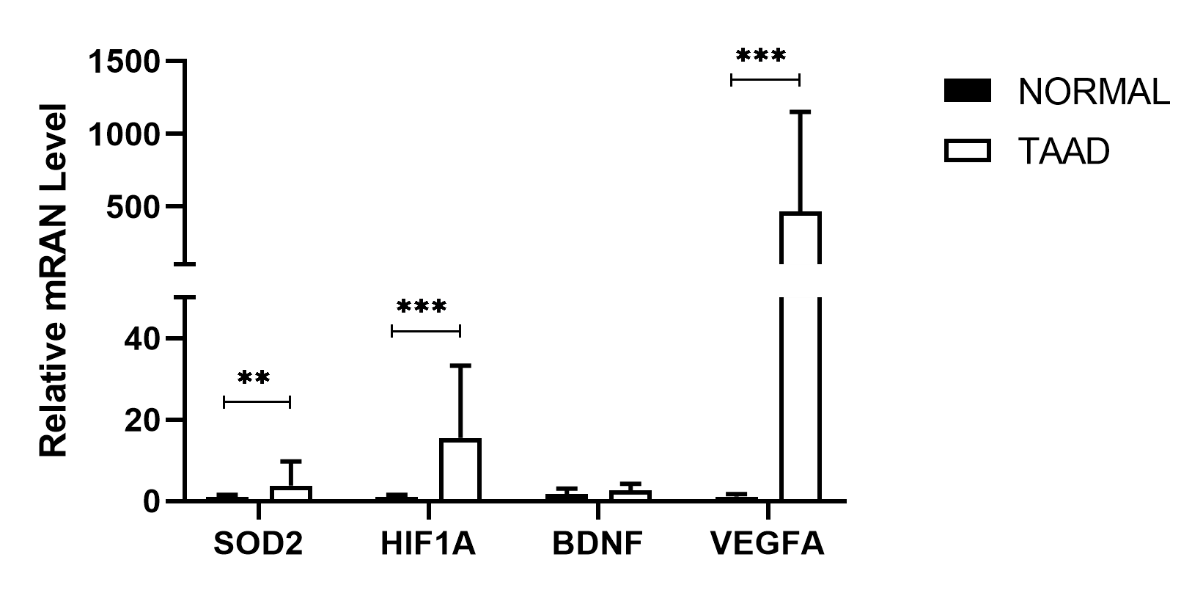


qPCR validation of hub genes. Data was expressed as mean ± SD. **p = 0.0017, ***p < 0.0001. Unpaired Student’s t-test was used to analyze the data.

Table S1

|  | **TAAD (n=7)** | **NORMAL (n=4)** | **P-value** |
| --- | --- | --- | --- |
| Age (years)  Male (%)  Height (cm)  Weight (kg)  BMI (kg/m^2^)  Aortic diameters (mm)  Smoking  Hypertension  Diabetes  Alcoholism  CKD  Stroke | 55.5±9.66  5 (71%)  169.9±8.8  78.4±17.3  27.1±5.4  57.0±3.6  4 (57%)  5 (71%)  0 (0%)  1 (14%)  0 (0%)  0 (0%) | 71.75±7.08  3 (75%)  167.8±7.3  72.8±10.3  25.7±1.8  ND  2 (50%)  4 (100%)  2 (50%)  1 (25%)  0 (0%)  0 (0%) | 0.02  1.00  0.71  0.59  0.67  --  1.00  0.49  0.11  1.00  1.00  1.00 |

BMI, Body mass index; ND, not detected.
